# Genetic and epigenetic regulation of cortactin (*CTTN*) by inflammatory factors and mechanical stress in human lung endothelial cells

**DOI:** 10.1042/BSR20231934

**Published:** 2024-09-16

**Authors:** Xiaoguang Sun, Belinda Sun, Saad Sammani, Steven M Dudek, Patrick Belvitch, Sara M. Camp, Donna Zhang, Christian Bime, Joe G.N. Garcia

**Affiliations:** 1 Department of Medicine, University of Arizona, Tucson, AZ, U.S.A.; 2 Department of Pathology, University of Arizona, Tucson, AZ, U.S.A.; 3 Department of Medicine, University of Illinois Chicago, Chicago IL, U.S.A.; 4 University of Florida, UF Scripps Research Institute, Jupiter, FL, U.S.A.; 5 College of Pharmacy, University of Arizona, Tucson, AZ, U.S.A.

**Keywords:** cortactin, inflammatory factors, promoter activity, transcriptional regulation

## Abstract

Rationale: Cortactin, an actin-binding cytoskeletal protein, plays a crucial role in maintaining endothelial cell (EC) barrier integrity and regulating vascular permeability. The gene encoding cortactin, *CTTN*, is implicated in various lung inflammatory disorders. Despite this, the transcriptional regulation of *CTTN* by inflammatory stimuli and promoter SNPs remains unexplored. Methods: We transfected human lung ECs with a full-length *CTTN* promoters linked to a luciferase reporter to measure promoter activity. SNP-containing *CTTN* promoter was created via site-directed mutagenesis. Transfected ECs were exposed to LPS (PAMP), TNF-α (cytokine), cyclic stretch (CS), FG-4592 (HIF-inducer), NRF2 (anti-oxidant modulator), FTY-(S)-phosphate (endothelial barrier enhancer), and 5′-Aza (demethylation inducer). Immunohistochemistry was used to assess cortactin expression in mouse lungs exposed to LPS. Results: LPS, TNF-α, and 18%CS significantly increased *CTTN* promoter activities in a time-dependent manner (*P*<0.05). The variant rs34612166 (-212T/C) markedly enhanced LPS- and 18%CS- induced *CTTN* promoter activities (*P*<0.05). FG-4592 significantly boosted *CTTN* promoter activities (*P*<0.01), which were partially inhibited by HIF1α (KC7F2) and HIF2α (PT2385) inhibitors (*P*<0.05). NRF2 activator Bixin increased *CTTN* promoter activities, whereas NRF2 inhibitor Brusatol reduced them (*P*<0.05). 5′-Aza increased *CTTN* promoter activities by 2.9-fold (*P*<0.05). NF-κB response element mutations significantly reduced *CTTN* promoter activities response to LPS and TNFα. FTY-(S)-phosphate significantly increased *CTTN* promoter activities in 24 h. *In vivo*, cortactin levels were significantly elevated in inflammatory mouse lungs exposed to LPS for 18 h. Conclusion: *CTTN* transcriptional is significantly influenced by inflammatory factors and promoter variants. Cortactin, essential in mitigating inflammatory edema, presents a promising therapeutic target to alleviate severe inflammatory disorders.

## Introduction

The pulmonary vascular endothelium serves as a semi-selective barrier between circulating blood and surrounding tissues, with endothelial cell (EC) integrity critical to tissue and organ function. The SARS-CoV-2/COVID-19 pandemic has resulted in an unprecedented number of patients with acute respiratory distress syndrome (ARDS) and has dramatically highlighted the role of EC loss of barrier integrity in ARDS pathobiology, with excessive vascular leakage and multiple vital organ failure driving ARDS mortality [1]. Disruption of vascular barrier integrity by inflammatory stimuli such as LPS, TNFα, eNAMPT, and IL-1β, and by excessive mechanical stress produced by mechanical ventilation, leads to hypoxemia, increased multi-organ failure and potential ARDS mortality [2–4].

The pathobiological mechanisms producing increased vascular permeability are incompletely understood but undoubtedly involve robust activation of the EC cytoskeleton, well recognized as critical to vascular barrier regulation and repair [5]. Inflammatory cell or mediator-induced activation of vascular barrier-disruptive signaling pathways, in combination with increases in reactive oxygen species (ROS), result in enhanced EC contractility, loosening of inter-endothelial junctions, formation of paracellular gaps, and development of profound vascular leakage and organ edema [5]. In addition, several cytoskeletal target genes harbor variants which contribute to the genetic basis of well-recognized health disparities in ARDS subjects of African descent, including cortactin [6–11].

The pleiotropic protein cortactin, encoded by the *CTTN* gene, is essential to cytoskeletal regulation of EC–matrix and cell–cell adhesion, vascular integrity, and permeability, which play key pathophysiological roles in lung inflammatory diseases [12–16]. In addition to EC barrier regulation, cortactin is centrally involvement in angiogenesis [17,18], EC apoptosis [19], and leukocytic diapedesis [20,21]. Cortactin is also a universal host cytoskeletal target of bacterial pathogens. By targeting cortactin, microbial pathogens exploit host cytoskeleton dynamics to enhance opportunistic increases in bacterial adherence, invasion, or intracellular motility [22]. *CTTN* is a cytoskeletal target gene whose coding variants (SNPs) also contribute to the genetic basis for observed ARDS health disparities in severe sepsis-induced ARDS and severe asthma [11,23,24] in African Americans (AA) and/or European Americans (EA). In two independent case-control samples of AA and EA severe asthma cohorts, *CTTN* variant rs3802780 were identified as consistently associated with susceptibility to severe asthma [24]. Our previous studies also demonstrated that *CTTN* coding SNP rs56162978 (S484N) significantly associated with mortality in patients with sepsis, and susceptibility of acute chest syndrome in sickle cell disease [11]. *In vitro*, S484N expression impairs wound closure, reduces EC barrier-promoting lamellipodia dynamics [23], and delays EC barrier recovery following thrombin-induced permeability [11]. *In vivo*, delivery of the *CTTN* WT transgene significantly attenuates vascular leak in ventilator-induced lung injury, whereas the *CTTN* S484N transgene failed to improve the lung injury [11].

Cortactin is a multi-domain protein that integrates multiple signaling pathways and cellular structures to regulate vascular permeability [16,25]. It can be phosphorylated at several key sites by c-Src, c-Abl, and other kinases that serve to modulate its interactions with other proteins and subcellular location [12,26,27]. For example, the endogenous phospholipid sphingosine-1-phosphate (S1P) rapidly improves EC barrier function by initiating a series of signaling events in which cortactin plays a critical role. These events include Rac1 activation, c-Abl-mediated phosphorylation of cortactin, cortactin recruitment to the EC periphery and interaction with myosin light chain kinase (MLCK), peripheral MLC phosphorylation and tension development, Arp2/3-mediated actin polymerization, lamellipodia formation, and intercellular gap closure [12,15,23,26]. In a study from another group, loss of cortactin leads to decreased adrenomedullin secretion, reduced cAMP-dependent Rap1 activation, increased ROCK-1 protein levels, increased MLC phosphorylation and contractile stress-fiber formation, and as a consequence increased endothelial permeability [14]. More recently, we confirmed that reduced cortactin expression is associated with increased levels of lung injury, while increasing cortactin expression exerts a protective role against inflammatory lung injury *in vivo* [11].

However, due to the high complexity of various causes and dramatical progress of ARDS, the relationship between cortactin and ARDS is also complicated and context-dependent. In other different animal models, cortactin was shown augmentation of acute lung injury (ALI) associated inflammatory states [21,28,29]. Cortactin deficiency is associated with increased vascular permeability *in vivo* and *in vitro*, but cortactin is required for leukocyte rolling, adhesion, and transmigration *in vivo* [28]. Cortactin promotes sepsis severity by supporting excessive neutrophil infiltration into the lungs in the cecal ligation and puncture (CLP) induced sepsis and indirect ALI model [21]. In another hemin- induced ALI model with sickle cell disease, cortactin loss protects against hemin-induced ALI [29].

Despite these studies highlighting the critical involvement of cortactin in acute inflammatory processes and vascular leak, much less information is available regarding the mechanistic regulation of the *CTTN* promoter activity by inflammatory factors. To date, most studies have focused on the effects of post-translation modifications (PTM) of cortactin, including ubiquitination [30], acetylation [17,31], phosphorylation [11,19], and glycosylation, which participate in pathophysiologic responses, such as altered permeability, inflammation, invasion, migration and degradation mechanisms, like autophagy and apoptosis [17,31–33]. We speculate that this rapid up-regulation of *CTTN* gene expression may reflect homeostatic mechanism in lung endothelium to regulate the magnitude of vascular leak during the early stages of inflammation. Indeed, the present study extends the prior reports to interrogate *CTTN* promoter activity regulation by ARDS-relevant inflammatory stimuli. *CTTN* transcriptional activity was regulated by the redox-sensitive transcription factor, NRF2, via an antioxidant response elements (ARE) that results in transcriptional enhancing cortactin expression. *CTTN* promoter is also under strong influence by hypoxia-inducible transcription factors, HIF-1α and HIF-2α. Furthermore, the *CTTN* promoter is regulated by LPS, excessive mechanical stress (mimicking ventilator-induced lung injury), and by a SNP that alters specific transcription factor binding and promoter DNA methylation. These findings are consistent with *CTTN's* contribution to inflammatory disease susceptibility with cortactin representing an attractive molecular target in complex lung disorders such as ARDS given the continued absence of FDA-approved ARDS pharmacotherapies [34].

## Methods

### Cell culture, cyclic stretch and reagents

Human pulmonary artery endothelial cells (EC) were obtained from Lonza (Walkersville MD) and cultured as described previously [12,35] in the manufacturer’s recommended endothelial growth medium-2 (EGM-2). Cells were grown at 37°C in a 5% CO_2_ incubator, and passages 6 to 9 were used for experiments with media changed one day before experimentation. For cyclic stretch (CS) studies, EC were plated on Bioflex collagen I type cell culture plates (FlexCell International, Hillsborough NC) and stimulated for 0–24 h at 18% CS as previously described [36] on the FlexCell FX-5000 System (FlexCell International), mimicking high tidal volume ventilation. For demethylation studies, EC were treated with 5-aza-2′-deoxycytidine (5′-Aza) (Sigma-Aldrich, St. Louis MO) for 24 h at indicated concentrations to inhibit DNA methyltransferase enzymes as we described previously [37].

### *In Silico* analysis and site mutagenesis of *CTTN* promoter

*Cis*-regulatory elements of *CTTN* promoter (NM_005231, NM_001184740) were analyzed by a database of transcription factor binding profiles (https://jaspar.genereg.net/) as previously described [35,38]. Plasmids containing GLuc-ON™ promoter reporter vector pEZX-PG04-*CTTN* promoter construct linked Gaussia luciferase (GLuc) gene and secreted alkaline phosphatase (SEAP) internal control were obtained from GeneCopoeia, Inc. (GeneCopoeia, Inc., Rockville, MD, USA). Gene mutagenesis was performed as previously described [39]. The DNA was modified by site-directed mutagenesis (QuikChange Lightning Multi Site-Directed Mutagenesis Kit, Agilent, CA) to generate fragments containing opposite alleles. Mutations of NF-κB binding sites were generated by deletion of core binding elements (GGGGG or TTCCC).

### Luciferase reporter gene assays

All constructs were transfected into ECs. After 48 h, transfected cells were exposed to indicated concentrations of FG-4592, Bixin, Brusatol, LPS, or to 18% CS for indicated period. As the cell medium contains secreted Gaussia luciferase and alkaline phosphatase (SEAP), Luciferase activities were measured by Secrete-Pair™ Dual Luminescence Assays Kit (GeneCopoeia Inc, MD) using the GloMax-Multi Detection System (Promega). Relative activities were expressed as the ratio of Gaussia luciferase to SEAP. Four to six independent transfections and duplicate luciferase assays were performed for each condition.

### Immunohistology

All animal procedures were approved by the Institutional Animal Care and Use Committee. After anesthetizing, mice were exposed to intratracheal LPS or PBS as described previously [40]. Briefly, four mice underwent instillation of LPS (Sigma-Aldrich, St. Louis, MO, U.S.A.; *Escherichia coli* 0127: B8, 0.1 mg/kg) in 40 µl of 0.9% sterile phosphate-buffered saline (PBS) into the lungs by cannulating the trachea through the vocal cords. The other four mice underwent installation with PBS alone. Lungs were harvested 18 h post-instillation of LPS or PBS. The cortactin proteins in mice lungs were detected by immunohistochemistry (IHC) staining with cortactin antibodies (mouse monoclonal IgG, sc-55579, Santa Cruz, Dallas, Texas, U.S.A.) at 1:50 dilution, and biotinylated secondary antibodies. The IHC image was analyzed, and positive staining of cortactin was quantified by ImageJ software as described previously [38,41].

### Statistical analysis

The ANOVA test was used for comparison of luciferase activities among different constructs. Otherwise, Student’s *t*-test was used, and the results are expressed as mean ± SEM. Statistical significance was defined at *P*<0.05 in all tests.

## Results

### Extrinsic inflammatory mediator bacterial endotoxin increases cortactin (*CTTN*) promoter activity in a time-dependent manner

**Figure 1:**
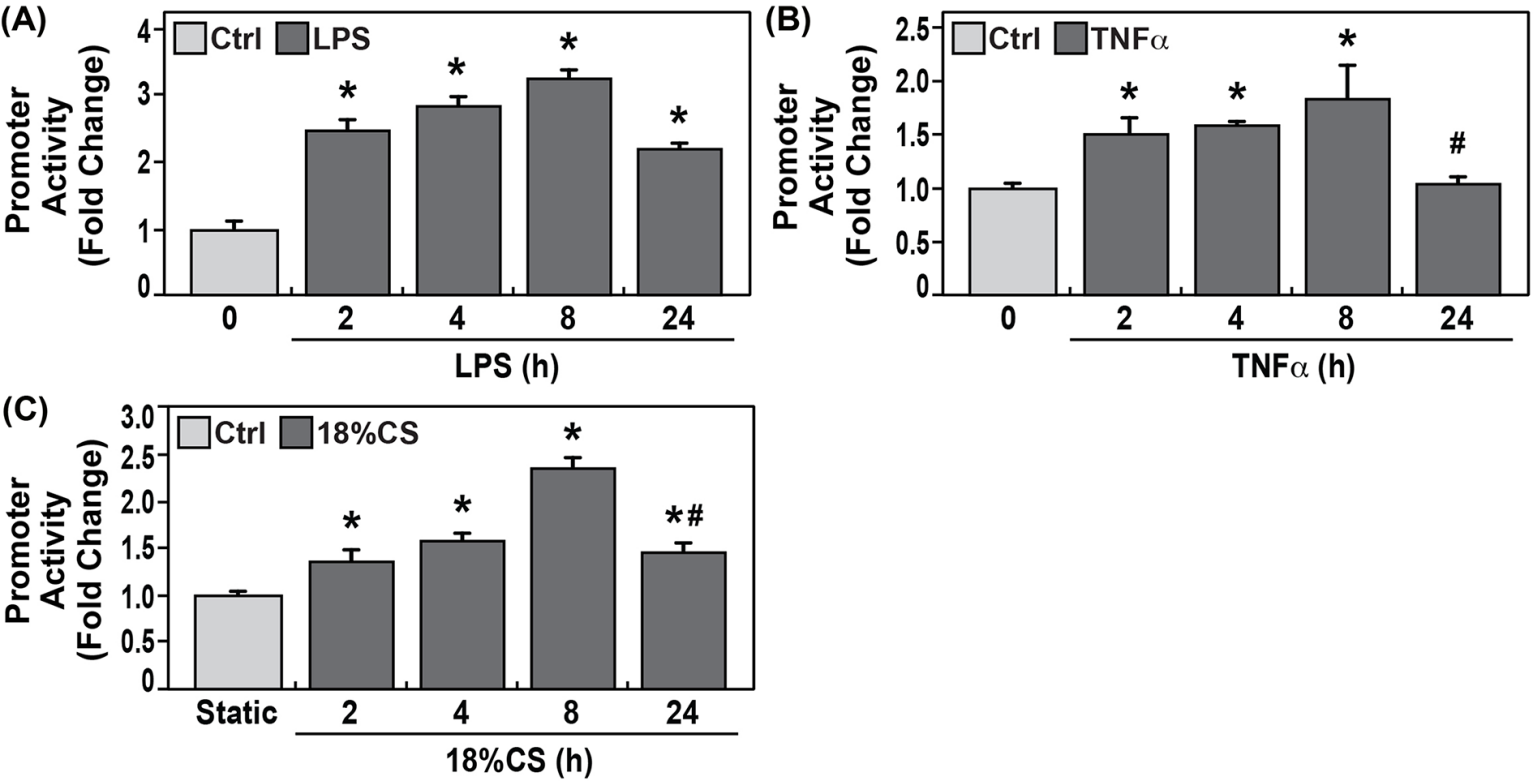
LPS, TNF-α, and excessive mechanical stress increases *CTTN* promoter activities in a time-dependent manner *CTTN* promoter activity reporter was constructed using 1.6 kb of the *CTTN* promoter (NM_005231) with the gene for secreted Gaussia Luciferase (GLuc) and secreted Alkaline Phosphatase (SEAP) (HPRM37676-PG04) from GeneCopoeia Inc. (Rockville, MD). Human pulmonary artery ECs were transfected with full-length *CTTN* promoter constructs and exposed to LPS 100 ng/ml (**A**), TNF-α (10 ng/ml) (**B**), or 18% cyclic stretch (**C**) for 0, 2, 4, 8, and 24 h. The supernatants were collected, and the luciferase activities of GLuc for *CTTN* promoter and AP activities for internal controls were measured by Secrete-Pair Dual Luminescence Assays (GeneCopoeia Inc.). The ratios of GLuc to SEAP in five groups were compared. The bar graph represents normalized relative luciferase activity by vehicle-treated control (*P<0.05 vs. control, *n* = 4 each group). Compared with controls, LPS significantly increased *CTTN* promoter activity at 2 h (2.5-fold), 8 h (3.3-fold), and 24 h (2.2-fold) (each *P<0.05, vs. 0 h, *n* = 4 each) (A). TNF-α (10 ng/ml) significantly increased *CTTN* promoter activities at 2 h (1.5-fold), 4 h (1.6-fold), and 8 h (1.8-fold) (each *P<0.05, vs. 0 h, *n* = 4 each) (B). 18% cyclic stretch significantly increased *CTTN* promoter activities at 2 h (1.4-fold), 4 h (1.6-fold), 8 h (2.4-fold), and 24 h (1.5-fold) (each *P<0.05 vs. 0 h, #*P*<0.05 vs. 8 h, *n* = 4 each) (C).

Lipopolysaccharides (LPS) are important contributors to the development and severity of ARDS/VILI [42]. *In silico*, potential LPS-induced *cis*-regulatory elements for NFκB, IRF3/7, and AP-1 binding sites were identified on the *CTTN* promoter (Table 1). We next explored the effects of LPS (100 ng/ml, 2, 4, 8, 24 h) on *CTTN* gene transcription activity in human lung ECs containing the *CTTN* promoter luciferase reporter. LPS caused a significant and sustained increase in *CTTN* promoter activity, starting as early as 2 h (2.5-fold), peaking at 8 h (3.3-fold) and gradually declining by 24 h (2.2-fold) (Figure 1A) (*P<0.05).

**Table 1:** *In silico* analysis of *cis*-elements of *CTTN* promoter

Transcription factors/CpG sites	Start (bp to TSS)	End (bp to TSS)	Predicted binding sequences
NFkB	-880	-870	AGGACTTCCCG
	-134	-124	GGGGGTCCGCC
	-284	-274	TCCTTCCCCCG
IRF3/7	-1230	-1217	TAGAAAGCTAAACT
AP-1	-475	-469	TGACTCA
	-33	-25	ACCGGAAGT
	-194	-184	CTACTTCCGGG
STAT3	-674	-664	CTGCCTGGAAA
STAT5	-410	-399	CCTCCAAGAAAG
HIF1/2	-308	-299	GCACGTGCGT
	-943	-936	AGGCGTGG
NRF2	-458	-448	GGGACATAGCA
	-33	-24	ACCGGAAGTA
CpG Island	-400	+1	

### Intrinsic pro-inflammatory cytokines significantly increase *CTTN* promoter activity

Tumor necrosis factor-α (TNFα) is an inflammatory early response factor in lungs and an important contributor to the development and severity of ARDS/VILI [42]. *In silico*, potential TNFα-induced *cis*-regulatory elements for NFκB binding sites were identified on the *CTTN* promoter (Table 1). PG04-*CTTN* promoter were transfected into HPAECs, and promoter activity was measured after exposure to TNFα (10 ng/ml) for 0, 2, 4, 8, or 24 h. TNFα significantly increased *CTTN* promoter activity with a peak at 8 h (∼1.8- fold) (*P<0.05), and this increase was significantly attenuated by 24 h (Figure 1B).

### Mechanical stress significantly increased *CTTN* promoter activity

As a life-saving intervention for ARDS, mechanical ventilation is also associated with ventilator-induced lung injury (VILI) [42]. *In silico*, potential *cis*-regulatory elements for NFκB and STAT3/5 binding sites were identified on the *CTTN* promoter (Table 1). We next analyzed *CTTN* luciferase reporter promoter activation in response to 18% cyclic stretch (18% CS) in lung ECs. 18% CS significantly increased *CTTN* promoter activity within 2 h (1.5- fold), reached the peak by 8 h (2.4-fold), and remained significantly elevated at 24 h (*P*<0.05 vs static) (Figure 1C).

### Regulation of *CTTN* promoter activities by a *CTTN* variant

**Figure 2:**
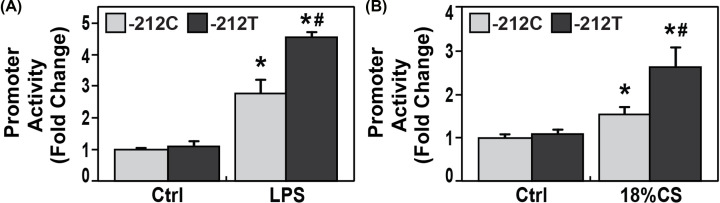
*CTTN* promoter variant rs34612166 (-212C/T) significantly augments LPS- and excessive mechanical stress-induced *CTTN* promoter activities rs34612166 (-212C/T) was identified to have lower frequency in sickle cell population (*P*<2 × 10^−7^) and predicted to alter transcription factor binding to *CTTN* gene and alter transcription regulation. *CTTN* promoter reporters containing rs34612166T (-212T) with GLuc and SEAP gene were generated from 1.6 kb *CTTN* promoter (NM_005231) reporters by site-directed mutagenesis. The constructs were transfected into ECs and exposed to LPS 100 ng/ml (**A**) or 18% cyclic stretch for 4 h (**B**). Compared with the promoter containing -212C, *CTTN* promoter with -212T demonstrated significantly higher activity upon exposure to LPS for 4 h (1.8-fold) (*P<0.05 vs. controls, #*P*<0.05 vs. -212C/LPS, *n* = 4 each) (A). Compared with the promoter containing -212C, *CTTN* promoter with -212T demonstrated significantly higher promoter activity upon exposure to 18% cyclic stretch for 4 h (1.7-fold) (*P<0.05 vs. controls, #*P*<0.05 vs. -212C/18%CS, *n* = 4 each) (B).

In a recent GWAS study, we identified one *CTTN* SNP rs34612166 (-212C to T) has significantly lower frequency in patients with sickle cell disease than healthy African Americans (*P*<2 × 10^−7^) (to be submitted separately). *In silico* analysis by PredictSNP2, the SNP is predicted to significantly alter *CTTN* gene function [43]. It was predicted to change several transcription factor recruitments to *CTTN* promoter by Genomatix (http://www.genomatix.de). By site-directed mutagenesis, we generated the *CTTN* promoters containing either rs34612166C (-212C) or rs34612166T (-212T) on Gaussia and SEAP luciferase reporter. The constructs were transfected into HPAEC and exposed to LPS 100 ng/ml or 18% CS for 4 h, followed by Dual Luminescence assay. There were no significant differences in basal promoter activity of the *CTTN* promoter containing -212T compared with -212C. However, the *CTTN* promoter containing -212T demonstrated significantly higher promoter activity than one containing -212C after exposure to LPS for 4 h (∼1.8-fold, #*P*<0.05) (Figure 2A). When EC were stimulated by 18% CS for 4 h, *CTTN* promoter containing -212T also demonstrated significantly higher promoter activity than one containing -212C (∼1.7-fold, #*P*<0.05) (Figure 2B).

### Up-regulation of the *CTTN* promoter activity by hypoxia-inducible factors

**Figure 3:**
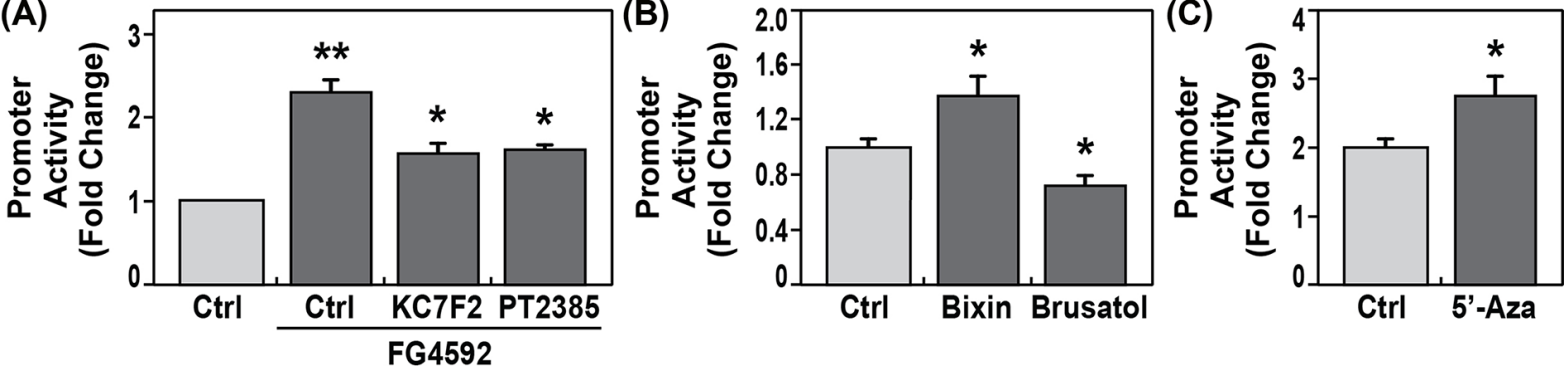
Hypoxia-inducible factors, nuclear factor erythroid 2–related factor 2 (NRF2) and demethylation up-regulate *CTTN* promoter activities *CTTN* promoter activity reporter constructs containing 1.6 kb *CTTN* promoter (NM_005231) with gene GLuc and SEAP were transfected into human pulmonary artery ECs exposed to HIF prolylhydroxylase inhibitor, FG-4592, or vehicle for 4 h, with or without HIF-1α inhibitor, KC7F2, or HIF-2α inhibitor, PT2385, respectively. Compared with vehicle, FG-4592 100 mM significantly increased *CTTN* promoter activity approximately 2.3-fold (**P<0.01 vs. vehicle, *n* = 4 each). Compared with FG-4592 alone, KC7F2 (40 μM) or PT2385 1 μM significantly attenuated effects of FG-4592 (*P<0.01 vs. FG-4592, *n* = 4 each) (**A**). To measure effects of NFR2, transfected ECs were stimulated by either NRF2 activator Bixin (4 μM) or NRF2 inhibitor Brusatol (4 nM) for 4 h. Compared with vehicle, Bixin significantly increased *CTTN* promoter activity (1.4-fold) (*P<0.05 vs. control, *n* = 4 each). Brusatol significantly decreased *CTTN* promoter activity (0.7-fold) (*P<0.04 vs. control, *n* = 4 each) (**B**). To detect the effects of demethylation on *CTTN* activity, ECs transfected with CTTN promoter were exposed to 5′-Aza for 24 h. Luciferase activities were measured using the Secret-Pair Dual Luciferase Assay System. Compared with controls, 5′-Aza significantly increased *CTTN* promoter activity (*P<0.05 vs. control, *n* = 4 each) (**C**).

Profound hypoxia is the hallmark of ARDS pathophysiology [44]. Hypoxia-inducible factors (HIF), HIF-1α and HIF-2α, are transcription factors activated by exposure to hypoxia with nuclear binding to HIF-response elements (HREs) [42]. *In silico*, potential hypoxia induced-*cis*-regulatory elements for HIF1/2 binding sites were identified on the *CTTN* promoter (Table 1). Both hypoxia and the HIF prolylhydroxylase (PHD) inhibitor, FG-4592, block the degradation of HIF-1α and HIF-2α to increase intracellular protein levels. Human ECs were transfected with *CTTN* promoter reporter, then treated with vehicle or PHD inhibitor FG-4592 (100 mM) for 4 h, with or without specific inhibitors for HIF-1α or HIF-2α, followed by measurements of dual luciferase activity. FG-4592 significantly increased *CTTN* promoter activities approximately 2.3-fold (**P<0.01 vs. vehicle). Specific HIF-1α inhibitor KC7F2 (40 μM) significantly attenuated effects of FG-4592 by 58% (*P<0.01 vs. FG-4592), while specific HIF-2α inhibitor PT2385 (1μM) significantly attenuated effects of FG-4592 by 54% (*P<0.01 vs. FG-4592) (Figure 3A). Thus, HIF-1α and HIF-2α contribute to temporal regulation of *CTTN* promoter activities.

### Up-regulation of the *CTTN* promoter activity by nuclear factor erythroid 2–related factor 2 (NRF2)

Oxidative stress and inflammation play crucial roles in the pathogenesis of ARDS, and NRF2 shows significant antioxidant and anti-inflammatory effects in different cells [42]. *In silico*, potential *cis*-regulatory elements for NRF2 binding sites were identified on the *CTTN* promoter (Table 1). In our study, *CTTN* promoter reporters were transfected into HPAECs and then stimulated with either NRF2 activator Bixin (4 μM) or inhibitor Brusatol (4 nM) for 4 h. Compared with vehicle, NRF2 activator Bixin significantly increased *CTTN* promoter activities by 1.4-fold, while NRF2 inhibitor, Brusatol, significantly decreased *CTTN* promoter activities by 0.7-fold (both *P<0.05 vs. control), respectively (Figure 3B). These studies indicate *CTTN* promoter activity is significantly regulated by the key antioxidant regulator, NRF2.

### Influence of DNA demethylation on *CTTN* promoter activities

*In silico*, a potential CpG island was identified on the proximal *CTTN* promoter region by GC-Profile 2.0 (http://tubic.org/GC-Profile2) (Table 1). We next addressed effects of epigenetic regulation on the *CTTN* promoter activity by transfecting ECs with *CTTN* promoter reporters and exposing them to the demethylation agent, 5-aza-2'-deoxycytidine (5′Aza) 5 μM for 24 h. 5′Aza significantly increased *CTTN* promoter activity by 2.9-fold (*P*<0.05, compared with control) (Figure 3C). These studies indicate strong epigenetic regulation of *CTTN* promoter activities.

### Endothelial cell barrier enhancer FTY-(S)-phosphate significantly increases *CTTN* promoter activity

**Figure 4:**
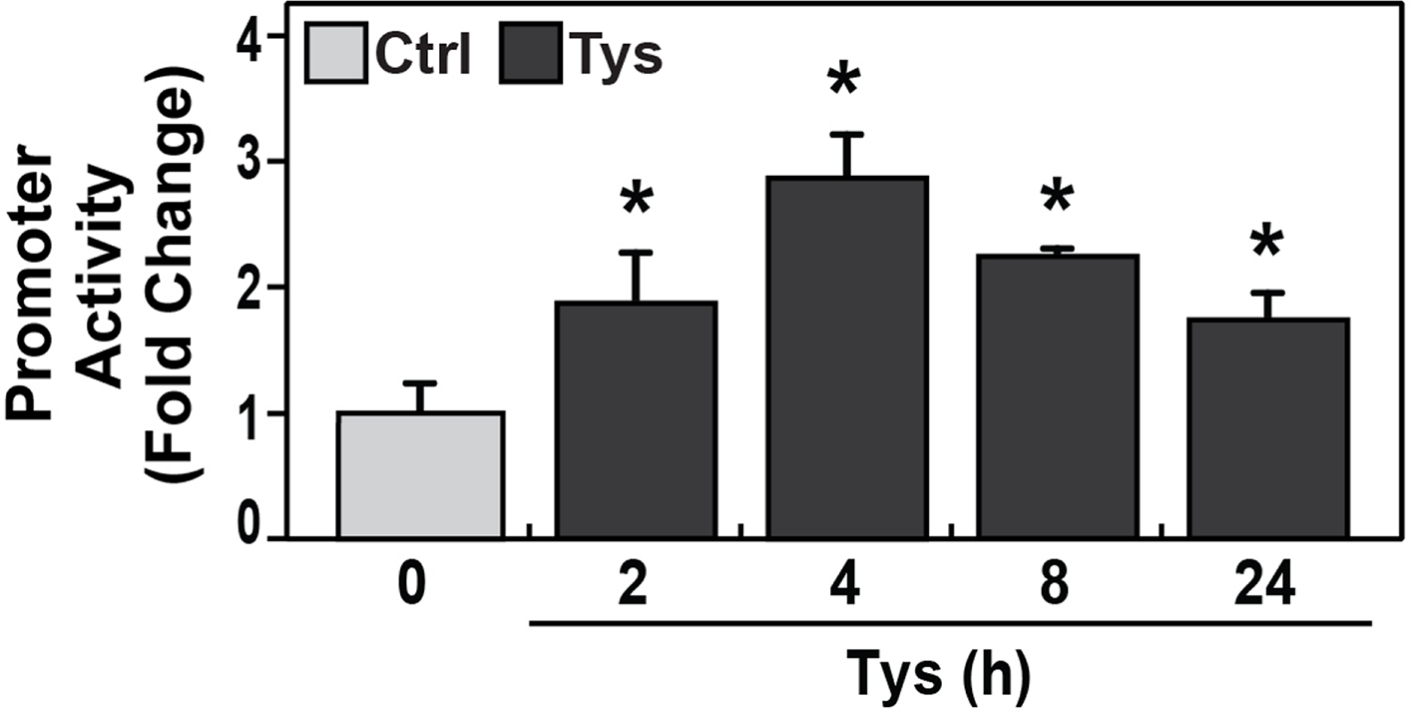
Endothelial cell barrier enhancer FTY-(S)-phosphate significantly increases *CTTN* promoter activity in a time-dependent manner *CTTN* promoter activity reporter was constructed using 1.6 kb of the *CTTN* promoter (NM_005231) with gene GLuc and SEAP were transfected into human pulmonary artery ECs exposed to FTY-(S)-phosphate (1 μM) for 0, 2, 4, 8, and 24 h. Compared with controls, FTY-(S)-phosphate significantly increased *CTTN* promoter activity at 2 h (1.9-fold), 4 h (2.9-fold), 8 h (2.3-fold), and 24 h (1.8-fold) (each *P<0.05, vs. 0 h, *n* = 6 each).

FTY-(S)-Phosphate (Tysiponate or Tys), the FTY720 (Fingolimod) analog, significantly induces EC barrier enhancement *in vitro* and reduces multiple indices of alveolar and vascular permeability in LPS-mediated murine model of ALI [45–47]. In this study, *CTTN* promoters were stimulated by Tys (1 μM) for 0–24 h. It is demonstrated that Tys significantly increased *CTTN* promoter activity by 2, 4, 8, and 24 h (*P<0.01 vs. 0 h). The increase started as early as 2 h, reached a peak by 4 h (∼2.9-fold) and sustained over 24 h (Figure 4).

### Transcription factor NF-κB mediated LPS- and TNFα- induced significant increases of *CTTN* promoter activity

**Figure 5:**
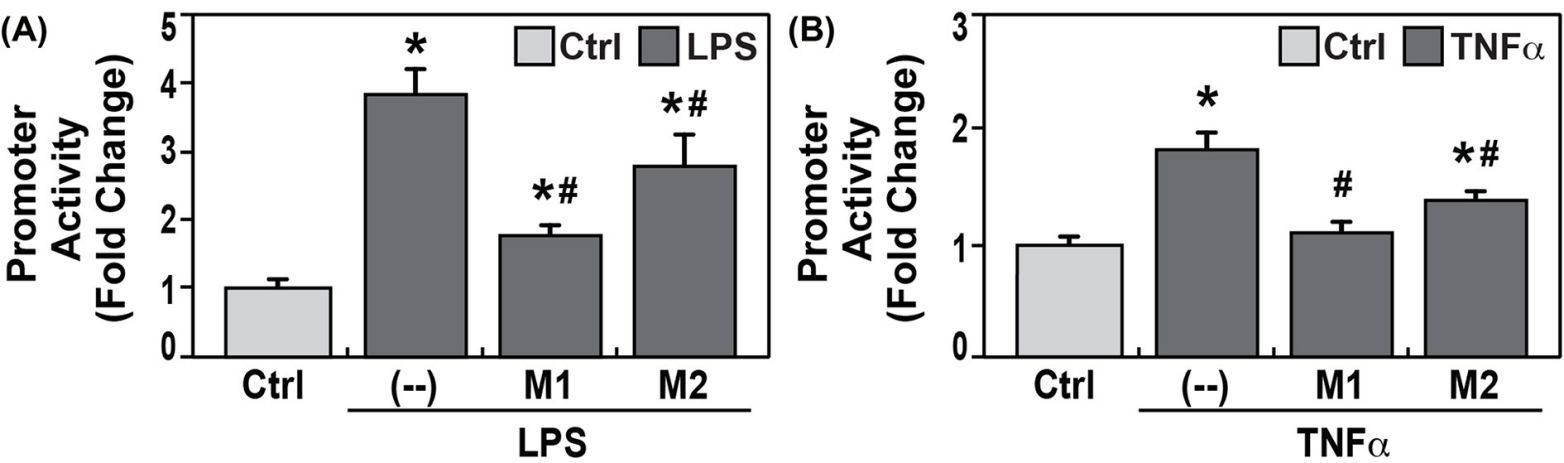
Transcription factor NF-κB mediated *CTTN* promoter response to inflammatory factor LPS- and TNF-α- by binding to NF-κB response elements *CTTN* promoter (NM_005231) with GLuc, and SEAP gene reporters underwent site-directed mutagenesis with deletion of core binding elements GGGGG or TTCCC for NF-κB binding to-134∼-124 bp (M1) and -880∼-870 bp (M2) sites at *CTTN* promoter, respectively, for disrupting NF-κB binding to *CTTN*. The constructs were transfected into lung HPACEs and exposed to LPS 100 ng/ml. All *CTTN* promoter activities were significantly increased by LPS (*P<0.01 vs. controls, *n* = 6 each), whereas the *CTTN* promoters with M1 or M2 demonstrated significantly reduced response to LPS, compared with wild-type promoter (#*P*<0.01, vs. LPS) (**A**). Next, the constructs were transfected into lung HPACEs and exposed to TNF-α 10 ng/ml for 4 h. *CTTN* promoter activities were significantly increased by TNF-α (*P<0.01 vs. controls, *n* = 6 each), whereas the *CTTN* promoters with M1 or M2 demonstrated significantly reduced response to TNF-α, respectively, compared with wild-type promoter (#*P*<0.01, vs. TNFα) (**B**).

NF-κB signaling is activated by numerous discrete stimuli and is required for induction of many inflammatory genes [48]. *In silico*, we identified three putative NF-κB binding sites on *CTTN* promoter (Table 1), including two sites with highest position weight matrix (PWM). By mutagenesis, we deleted core sequence for NF-κB binding at these two sites (-134∼-124 bp: M1; -880∼-870 bp: M2) and disrupted the NF-κB recruitments to the promoter. The promoter activity assay with transfected wild-type and mutated promoter demonstrated that disrupting NF-κB binding to the promoter both significantly attenuated LPS-induced robust increases of *CTTN* promoter activities, compared with wild-type (0.46-fold/M1 and 0.72-fold/M2, #*P*<0.01 vs. LPS), with more significant for M1 site mutation (Figure 5A). It is also demonstrated that disrupting either NF-κB binding sites significantly attenuated TNFα- induced robust increases of *CTTN* promoter activities, compared with wild-type (0.60-fold/M1 and 0.76-fold/M2, #*P*<0.01 vs. TNF), with more significant for M1 site mutation (Figure 5B).

### Endotoxin LPS significantly increased cortactin protein expression *in vivo*

**Figure 6:**
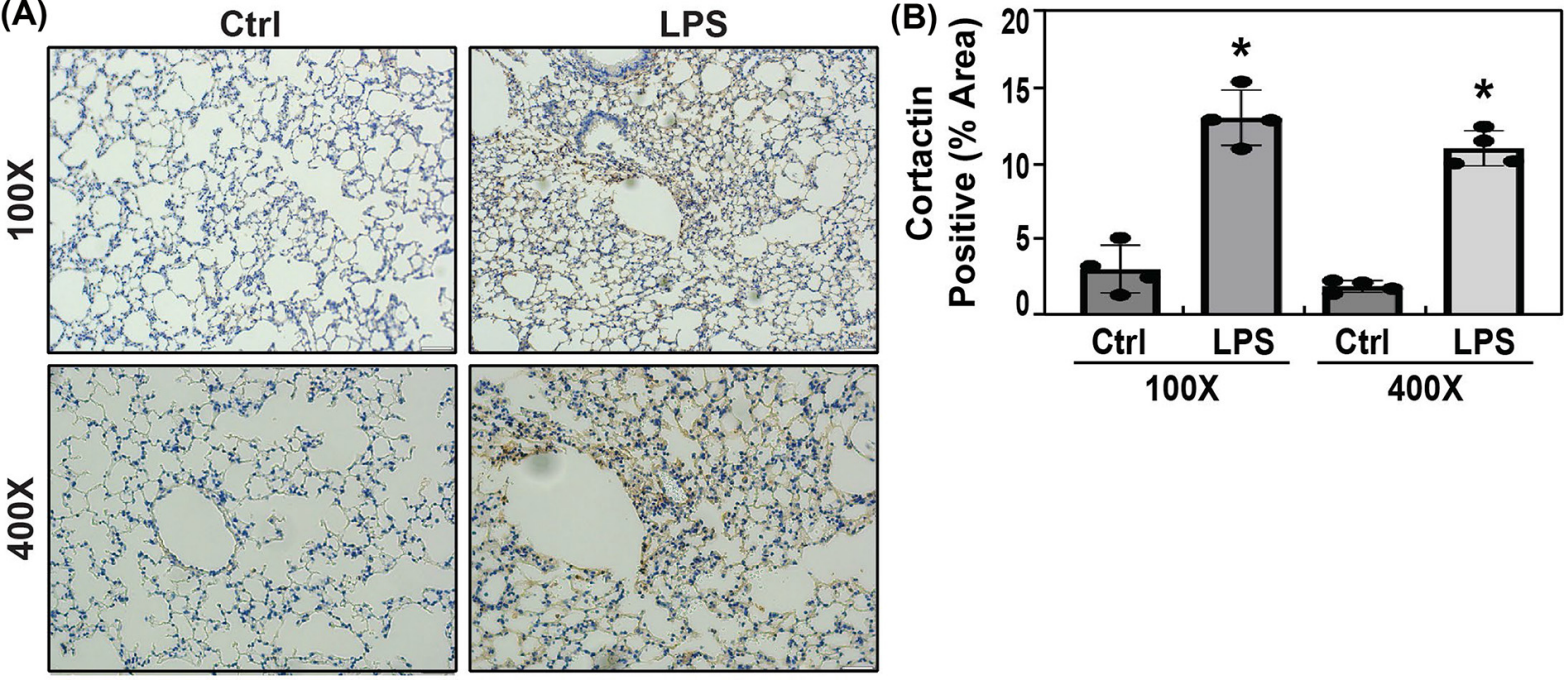
Endotoxin LPS significantly increased cortactin protein expression *in vivo* Mice were anesthetized by ketamine and xylazine, and intra-tracheal (I.T.) injected with LPS (*E. coli* 0127: B8, 0.1 mg/kg, Sigma-Aldrich) (*n*=4), or PBS (controls) (*n*=3). After 18 h, mice lungs were harvested, and lung tissue sections were stained. The cortactin proteins in mice lungs were detected by immunohistochemistry (IHC) staining with cortactin antibodies (mouse monoclonal IgG, sc-55579, Santa Cruz Biotechnology) at 1:50 dilution, and biotinylated secondary antibodies. IHC staining of LPS-exposed lung tissues exhibited increases in cortactin immunoreactivity (brown), compared with controls (magnification: ×100, ×400) (**A**). The IHC image was analyzed, and positive staining of cortactin was quantified by ImageJ software. The areas of cortactin protein positive staining were significantly expanded in mice lungs after exposure to LPS, compared with vehicle PBS controls (*P<0.01, vs. controls,* n* = 4 each, with magnification ×100, ×400) (**B**).

To further explore the post transcriptional regulation of cortactin proteins under inflammatory conditions, we detected cortactin proteins in inflammatory lungs by IHC after mice exposed to intra-tracheal moderate concentration of LPS (0.1 μg/ml) for 18 h. The IHC results demonstrated that cortactin proteins were ubiquitously increased in inflammatory mice lungs after exposure to LPS, compared with vehicle (PBS) controls (Figure 6A,B).

## Discussion

The incidence of ARDS among non-survivors of COVID-19 is 90% [49]. Despite improved understanding of the pathophysiology of ARDS, the underlying mechanisms for the injurious effects of inflammatory processes in the setting of ARDS remain unclear and effective pharmacotherapies have not yet emerged. We previously identified cortactin as a critical mediator for the restoration of pulmonary vascular barrier integrity which is profoundly lost during the initial stages of ARDS. Cortactin interacts with endothelial nmMLCK to regulate cytoskeletal rearrangement and endothelial barrier function [12,27] with disease-associated cortactin variants adversely affecting restoration of lung EC barrier function [11,23]. In short, this prior work identified cortactin as a critical lung cytoskeletal effector protein intimately involved in regulating the repair phase of inflammation-induced ARDS and VILI pathologic processes including increased lung vascular permeability.

In the present study, we explored the underlying molecular mechanisms involved in regulation of *CTTN*, the gene coding cortactin, in the settings of ARDS- and VILI-related inflammatory processes. We demonstrated that LPS, excessive mechanical stress, cytokines, hypoxia-inducible factors, antioxidant transcription factors, DNA demethylation, and a *CTTN* SNP, identified in a GWAS of a sickle cell disease cohort, each significantly influence *CTTN* promoter activity.

First, we demonstrated that the ARDS-relevant bacterial endotoxin (LPS) significantly increased *CTTN* transcription activities in a time-dependent manner. Since pathophysiological pathways of LPS-induced acute lung injury occur in a time-sensitive manner [50], with most transcription factor responses to LPS stimuli occurring in 24 h [51], we tested the effects of LPS on *CTTN* promoter/transcription activities over a 24 h period. The results indicate that the regulatory effects of LPS on *CTTN* promoter activity begin quickly within 2 h and are sustained for 24 h. While we did not identify the exact transcription factors (TFs) involved in *CTTN* regulation, *in silico* analysis of the *CTTN* promoter region indicate the presence of binding sequences for known transcription factors involved in LPS transcriptional regulation in ECs or mononuclear cells, including a central regulator of EC dysfunction, NFκB [52–55], the anti-inflammation factor, IRF3/7 [51,56,57] and the multi-functional TF, AP-1 [58,59].

*CTTN* transcription activity was also significantly regulated in a time-dependent manner by the intrinsic pro-inflammatory cytokine TNFα. TNFα-responsive promoter regions are known to contain *in silico* binding sequences for TFs involved in EC transcriptional regulation, including NFκB [53–55]. NF-κB signaling is activated by numerous discrete stimuli and is a master regulator of the inflammatory response to pathogens and cancerous cells [60]. Indeed, multiple potential binding sites of NFκB were identified in the *CTTN* promoter. Prior work indicated that TNFα induces cortactin redistribution in ECs to the site of PMN transmigration [20], with cortactin also involved in pronounced NF-κB activation and IL-8 release upon infection [61]. The current study demonstrates that NF-κB directly mediated increases of *CTTN* promoter activity response to inflammatory factor LPS and TNFα, and NF-κB binding to its two response elements (M1 and M2) on *CTTN* promoter are critical for these responses.

Given the essential contribution of excessive ventilator-induced mechanical stress to ARDS mortality [62], we explored and detected *CTTN* promoter/transcription responses to be heavily influenced by 18% CS thereby providing a mechanistic link between increased vascular permeability and VILI while corroborating additional genetic mechanisms of *CTTN* regulation [11]. Our previous studies in EC identified the STAT family of transcription factors, STAT5a/b and STAT3, to be significant contributors to regulation of mechanical stress-induced transcriptional activity, similar to what we have described for promoter responses to the critical damage-associated molecular pattern proteins (DAMPs), *NAMPT* [4,35,63–65], and *HMGB1* [66], each serving as key DAMPs in ARDS and as viable ARDS/VILI therapeutic targets. *In silico* analysis confirmed TF response elements for STAT3 and STAT5 to be present within the *CTTN* promoter.

Our study also addressed the influence of the *CTTN* promoter SNP, rs34612166 (-212C/T), on *CTTN* promoter activity and found -212C/T robustly augments the increased *CTTN* promoter activity in response to inflammatory factor LPS and excess mechanical stress 18% CS. *In silico* analysis suggests that rs34612166 alters TF binding sites in the *CTTN* promoter for AP-2, CTCF, INSM, Smad1/5 and Sp1, each implicated in regulation of lung inflammation and endothelial function (www.genomatix.de).

Hypoxemia is a critical pathological hallmark of ARDS/ALI and is a potent stimulus for amplification of ARDS inflammatory cascades via the hypoxia-inducible transcription factors, HIF-1α and HIF-2α [67–70]. Both HIF-1α and HIF-2α are multifunctional in ARDS. HIF-1α is responsible for LPS-induced IL-1β expression [71] and increases in other cytokines (TNF, IL-12) and VEGF [72]. HIF-1α also paradoxically reduces ventilator-induced ALI [73] by transcriptional regulation of A2B receptors [74]. We have shown HIF-1α and HIF-2α involvement in *NAMPT* promoter activities and protein expression [72,75,76], and now demonstrate that HIF-1α and HIF-2α accumulation (elicited by PHD2 inhibition) to significantly increase *CTTN* promoter activity. These results suggest that HIF1α/2α and cortactin gene interactions contribute to the resolution of ARDS/ALI [77–79].

The Nrf2 TF is essential for protection against acute pulmonary injury by up-regulating antioxidant gene expression to decrease oxidative stress and inflammation [80,81]. Nrf2-deficient mice demonstrated increased ARDS severity with enhanced lung inflammation [82]. In addition to induction of antioxidant genes [83], NRF2-mediated protection in models of ARDS/ALI also involves down-regulation of pro-inflammatory mediators [81]. In our previous study, NRF2-driven repression of *MYLK* expression, encoding nmMLCK, attenuated inflammatory lung injury [84]. Here we demonstrated that *CTTN* promoter activity was significantly enhanced by NRF2 activation and repressed by NRF2 inhibition. These results suggest NRF2 involvement in repair phase of ARDS via synergetic effects on repression of *MYLK* and promotion of *CTTN* transcription.

In previous studies, we have found that FTY-(S)-Phosphate (Tysiponate or Tys), the FTY720 (Fingolimod) analog, significantly induces EC barrier enhancement *in vitro* and reduces multiple indices of alveolar and vascular permeability in LPS-mediated murine model of ALI [45–47]. The current study confirmed that transcription activities of *CTTN* were significantly augmented by FTY-(S)-phosphate in a time dependent manner, which might be involved in this agent-induced endothelial barrier enhancement.

Previous studies suggest aberrant DNA methylation of lung tissues may be involved in the pathophysiology of LPS-induced ALI/ARDS [85] via hypomethylation of early response genes in [73]. We have previously demonstrated excessive mechanical stress and LPS to reduce *NAMPT* promoter DNA methylation to increased gene transcription [37]. *In silico*, multiple CpG sites were identified on the *CTTN* promoter, especially a large CpG island covering -400 bp to +1 TSS. Indeed, our current studies with *CTTN* indicate epigenetic regulation of the *CTTN* promoter via demethylation that significantly enhances promoter activities. In contrast, recent genome-wide DNA methylation meta-analysis identified the *CTTN* gene as exhibiting a significant increase in DNA methylation among current versus never smokers for COPD [86].

In summary, we examined molecular, genetic, and epigenetic mechanisms involved in regulation of the *CTTN* gene under lung inflammatory conditions relevant to ARDS/VILI pathobiology. Our data indicate that extrinsic insults (LPS), intrinsic factors (TNFα), and mechanical hyperventilation all increased *CTTN* promoter/transcription activity within 24 h and in a time-dependent manner and are enhanced by a *CTTN* promoter variant. Furthermore, hypoxemia-inducible factor HIF1α/2α, antioxidant response factor NRF2, and demethylation all significantly increased *CTTN* promoter/transcription activity. Since cortactin can be activated by external stimuli to promote polymerization and rearrangement of the actin cytoskeleton [11,12,16], we speculate that these rapid *CTTN* promoter response may reflect the homeostatic repair mechanisms within lung endothelium during the early stages of inflammation [11,23]. However, as mentioned in the introduction, in different reports, cortactin was shown to be protective or augment ALI/ARDS. The roles of cortactin in ARDS likely depend on various factors, including experiment models and regulatory pathways, and the stage and severity of ARDS [11–16,21,23,28,29]. Further research is needed to clarify the precise role of cortactin in ARDS and to determine how it might be targeted for therapeutic interventions.

Limitations of this study include the absence of more detailed characterization of the specific inflammation-related TFs (including HIF1/2 and NRF2) involved in *CTTN* regulation. A series of changes and fluctuations of protein levels of cortactin during the different stages of ARDS (early, middle and late stages) were not determined with the cortactin protein known to be strongly regulated by post-translational modifications such as phosphorylation and ubiquitination during lung inflammation [11,16,30]. However, despite these limitations, our studies of *CTTN* promoter activity in ECs by the ARDS inflammatory stimuli and by ARDS transcription factors further confirm *CTTN* and its coding protein, cortactin, as attractive therapeutic targets to significantly attenuate inflammation-induced vascular permeability and inflammatory lung injury during ARDS and VILI [11–13,16,87,88].

## Clinical perspectives

- The genetic and epigenetic factors that influence the regulation of the *CTTN* promoter encoding cortactin, an important cytoskeletal protein and contributor to the severity of asthma and the ARDS, are unknown.
- Extrinsic and intrinsic inflammatory factors, such as LPS, excessive mechanical stress, cytokines, hypoxia, and oxidants were each found to significantly increase *CTTN* promoter activity with significant influence of a promoter variant.
- Up-regulation of *CTTN* promoter activity may be a compensatory cellular response to inflammatory injury signals and representing a potential therapeutic strategy.

## Data Availability

All supporting data are included within the main article and its Supplementary Files.

## Open Access

Open access for this article was enabled by the participation of University of Florida in an all-inclusive *Read & Publish* agreement with Portland Press and the Biochemical Society under a transformative agreement with Individual.

## Abbreviations

ALI: acute lung injury
ARDS: acute respiratory distress syndrome
CLP: cecal ligation and puncture
DAMP: damage-associated molecular pattern protein
HIF: hypoxia-inducible transcription factor
IHC: immunohistochemistry
NRF2: nuclear factor erythroid 2–related factor 2
PWM: position weight matrix
S1P: sphingosine-1-phosphate
TF: transcription factor
TNFα: tumor necrosis factor-α
VILI: ventilator-induced lung injury
